# Stigmatization of severe mental illness in India: Against the simple industrialization hypothesis

**DOI:** 10.4103/0019-5545.37320

**Published:** 2007

**Authors:** Sushrut Jadhav, Roland Littlewood, Andrew G. Ryder, Ajita Chakraborty, Sumeet Jain, Maan Barua

**Affiliations:** Centre for Behavioural and Social Sciences in Medicine, London, UK; * University College London, London, UK; ** Department of Psychology, Concordia University, Montreal, Canada; *** University of Calcutta, Kolkata, West Bengal, India; **** Dibrugarh University, Dibrugarh, Assam, India

**Keywords:** Ethnography, industrialization hypothesis, rural India, severe mental illness, stigma

## Abstract

**Background:**

Major international studies on course and outcome of schizophrenia suggest a better prognosis in the rural world and in low-income nations. Industrialization is thought to result in increased stigma for mental illness, which in turn is thought to worsen prognosis. The lack of an ethnographically derived and cross-culturally valid measure of stigma has hampered investigation. The present study deploys such a scale and examines stigmatizing attitudes towards the severely mentally ill among rural and urban community dwellers in India.

**Aim:**

To test the hypothesis that there are fewer stigmatizing attitudes towards the mentally ill amongst rural compared to urban community dwellers in India.

**Materials and Methods:**

An ethnographically derived and vignette-based stigmatization scale was administered to a general community sample comprising two rural and one urban site in India. Responses were analyzed using univariate and multivariate statistical methods.

**Result:**

Rural Indians showed significantly higher stigma scores, especially those with a manual occupation. The overall pattern of differences between rural and urban samples suggests that the former deploy a punitive model towards the severely mentally ill, while the urban group expressed a liberal view of severe mental illness. Urban Indians showed a strong link between stigma and not wishing to work with a mentally ill individual, whereas no such link existed for rural Indians.

**Conclusion:**

This is the first study, using an ethnographically derived stigmatization scale, to report increased stigma amongst a rural Indian population. Findings from this study do not fully support the industrialization hypothesis to explain better outcome of severe mental illness in low-income nations. The lack of a link between stigma and work attitudes may partly explain this phenomenon.

Major international studies suggest that schizophrenia has better prognosis in low-income nations and in rural settings.[1–3] The industrialization hypothesis has been advanced to explain this differential outcome[4] but remains untested. Briefly, this hypothesis argues that industrial economies and attendant life styles lead to poor support, intolerance, rejection, isolation, segregation and institutionalization of the severely mentally ill. The value placed on the autonomous individual in industrialized settings therefore accentuates social extrusion of the chronic mentally ill patient who assumes personal responsibility for the illness. In consequence, prognosis worsens in urban industrialized settings. Attempts to test such hypotheses have until recently been hampered due to a lack of culturally valid, ethnographically derived instruments.[5].

Stigma has been argued to be a major determinant of outcome of severe mental illness across cultures.[5] Studies on stigma and mental illness in the Indian setting have focused both on measurement of stigma and on locally important sociocultural factors shaping stigma.[6–9] Research at the National Institute of Mental Health and Neurosciences (NIMHANS), Bangalore, focused on the cultural dimension and cross-cultural comparison of stigma related to depression. This landmark study measured illness experience, symptom prominence and indicators of stigma among 80 outpatients from mainly urban backgrounds using the Explanatory Model Interview Catalogue (EMIC).[7] In addition, patients were clinically assessed on the Structured Clinical Interview for DSM-IIIR and the Hamilton Depressive Rating Scale. The EMIC approach is unique in that it facilitates clarifying the nature of relationship between stigma ratings, symptoms, narratives of suffering and clinical features. The study concluded, amongst other findings, that patients reporting somatic symptoms had lower stigma scores than those reporting psychological symptoms.[7] Qualitative analysis revealed that depressive symptoms were perceived as socially disadvantageous as these may affect marriage and social status. Utilizing databases from London and Bangalore, the authors subsequently compared patients presenting for the first time with a clinic diagnosis of ICD-9 Depressive Neurosis at both sites. They found higher levels of self-perceived stigma in the London sample.[6]

Researchers at the Schizophrenia Research Foundation (SCARF) in Chennai examined perceptions of stigma among caregivers in a sample of 159 urban patients attending outpatient clinic and fulfilling DMS-IV criteria for schizophrenia.[8] The study deployed the Family Interview Schedule, a subsection of the International Study of Schizophrenia.[10] Findings suggested that female sex of the patient and a younger age of both patient and caregiver were associated with greater stigma. An interesting conclusion that emerged from analyzing illness attributes of study subjects was “a lack of an explanation” for the illness among the high-stigma group of families.

These studies have utilized urban samples and addressed stigma among affected persons (i.e., caregivers and patients). Numerous other studies[11–13] have addressed public attitudes towards mental illness. These publications are not reviewed; they are unrelated to the objectives of this paper. The approach used in this research includes both a rural-urban comparison and an examination of what constitutes stigma amongst non-affected people in the Indian general population. This research hypothesized that rural Indian respondents would express less stigmatization, together with a more tolerant attitude, towards severely mentally ill compared to their urban counterparts. This would be in keeping with the industrialization hypothesis, which the study sought to test.

## MATERIALS AND METHODS

This study deployed a recently developed ethnographically derived stigmatization questionnaire (SQ) comprising 24 items and a 4-point response scale (ranging from “Yes, agree very much” to “No, not at all.”[5,14] Rather than present respondents with a vignette that has named psychiatric categories, the instrument uses a short vignette in lay terminology (see Appendix). The variants of the vignette were derived simultaneously in three different languages (Bengali, English and Sinhalese). Then, the English draft was translated and back-translated by a number of professional collaborators working in different countries and with different linguistic and ethnic groups. A consensus was arrived at with regard to a particular account of a young man who, from the point of view of the project's psychiatric collaborators, could be described as having a schizophrenic illness which responded partly to treatment. The history, development and psychometric evaluation of the SQ are described elsewhere.[5,14]

The Hindi, Assamese and Bengali versions of the SQ were administered to a random community sample of 291 (*N* Rural = 108, *N* Urban = 183) non-affected Indian respondents in Hindi at Dadhika village, Kanpur district, Uttar Pradesh state (rural); Assamese at Mohpara village, Golghat district, Assam state (rural); and Bengali at Kolkata city, West Bengal state (urban). These sites were chosen based on the researchers' prior knowledge of the local communities and pragmatics of access to the local population.

## RESULTS

Rural respondents had a mean age of 37.7 years (SD = 14.2) and had 12.7 years (SD = 3.2) of formal education, whereas urban respondents had a mean age of 34.9 years (SD = 13.7) and had 11.0 (SD = 5.5) years of formal education. Using Welch's *t*-test to account for heterogeneity of variance, the rural sample had significantly more years of formal education than did the urban sample, *t*′(277.58) = 3.21. Rural-urban differences for dichotomous variables are shown in Table 1. There were significant differences in the ratio of the number of manual workers to the number of nonmanual workers and in the ratio of individuals who had fathers who were manual workers versus those who had fathers who were nonmanual workers. In both cases, manual workers were more common in the rural sample.

**Table 1:** Comparison of rural and urban sample on categorical demographics

	Sex		Marital status		Occupation		Father's occupation		Family history of mental illness	
					
	Male	Female	Not married	Married	Manual	Non-manual	Manual	Non-manual	Yes	No
Rural	73	35	38	70	55	53	87	19	40	67
Urban	112	71	79	104	44	139	67	116	52	129
X^2^	X^2^(1) = 1.20		X^2^(1) = 1.80		X^2^(1) = 21.87*		X^2^(1) = = 55.74*		X^2^(1) = 2.32	

* *P* < 0.05

### Properties of the composite stigma score in the rural sample

Psychometric properties of the composite stigma score in seven samples, including the urban sample used here, are described in detail elsewhere.[14] Put briefly, the scale has acceptable reliability and essential unidimensionality in all seven samples. The composite stigma score was also sufficiently reliable in the Indian rural sample, a = 0.72. Cross-cultural measurement requires that scales have equivalent meanings in the cultures under consideration. The best evaluation of equivalence involves Item Response Analysis, a technique that unfortunately requires very large sample sizes. For these data, we instead tested for uniform and non-uniform differential item functioning across the two samples using ordinal regression. Following Zumbo[15] individual items served as dependent variables, and predictors were added in three steps: (1) total score; (2) total score + cultural group; (3) total score + cultural group + (total score × cultural group). A significant X^2^ difference, accompanied by a large change in the Nagelkerke *R*^2^ (i.e., >0.13) between steps one and two or between steps two and three, indicates differential item functioning - the item has different properties in the two samples, suggesting that participants responded to the items in different ways.

Evidence of differential item functioning was found for only one item - “Should he stay in hospital for his whole life?” The reliability analysis had previously shown that the relation between this item and the total score was much lower in the rural sample than in the urban sample. All analyses conducted in this study were therefore repeated with this item dropped from the composite stigma score. In every case, the pattern of results was the same and the effect sizes were either the same or slightly higher. Results from the full composite stigma scale have been detailed elsewhere.[14]

### Rural-urban differences in individual and composite stigma indicators

Rural-urban differences in individual stigma indicators and the composite stigma score are shown in the top panel of Table 2. At the item level, six items showed significant differences in the direction of greater stigma in the rural sample; no items showed the reverse pattern. Similarly, the composite stigma score indicated a greater degree of overall stigma in the rural sample. Significantly differing items and the total scores had moderate effect sizes somewhat more than one-half of a standard deviation. Given the observed cultural differences in education level and occupation, this latter finding was further tested using Analysis of Covariance (ANCOVA), with (a) rural *vs*. urban and manual *vs*. nonmanual occupation as independent variables; (b) the composite stigma score as the dependent variable; and (c) education level as a covariate. Results showed a significant interaction effect, revealing that the rural-urban difference in stigma can be explained by high levels of stigma in the rural manual laborers, *F* (1.276) = 7.91, *P* < 0.05; rural nonmanual laborers had stigma scores equivalent to those of individuals in the urban sample.

**Table 2:** Comparison of rural and urban samples on stigma questionnaire

Stigma questions	Rural		Urban		*t*	*d*
				
	M	SD	M	SD		
Would you be frightened if this man came to live next door to you?	1.86	1.00	1.55	0.88	2.69*	0.33	
Do you think he will get ill again even if he takes the doctor's medicine?	2.66	1.02	1.80	0.88	7.33*	0.90
Should he take part in meetings of his family which are to make important decisions? (-)	2.01	1.11	2.14	1.18	−0.92	0.11
Would you be happy if he married your sister? (-)	3.43	0.99	3.20	1.02	1.84	0.23
Could he suddenly become physically violent?	2.75	1.04	2.71	1.08	0.31	0.04
If he was your brother would it be important not to let other people know that he had been ill, to avoid shame for your family?	1.88	1.17	1.73	1.04	1.08	0.14
Should the doctors tell him not to have any children in case he passes the illness on to them?	1.78	1.11	1.96	1.15	−1.30	0.16
Should the doctors have let him out of hospital? (-)	2.60	1.23	1.89	1.17	4.82*	0.59
Would it be wise for this man to inherit his parents' property? (-)	1.98	1.12	1.50	0.93	3.76*	0.47
Would you be happy if this person became the teacher of your children? (-)	3.10	1.09	2.89	1.12	1.62	0.19
Will he be able to return to a completely normal life? (-)	1.58	0.78	1.49	0.65	1.10	0.13
Should he stay in hospital for his whole life?	1.52	0.65	1.13	0.49	5.38*	0.68
Would you eat food which he has cooked? (-)	2.45	1.20	1.63	0.90	6.17*	0.77
Would you avoid talking to him if possible?	2.19	1.06	1.61	0.88	4.73*	0.70
Composite Stigma Score	2.27	0.48	1.95	0.51	5.44*	0.65
Would you be content if he was to work together with you in your workplace? (If you do not have a job, answer as if you did.) (-)	2.74	1.04	2.61	1.17	0.97	0.12
If your local hospital opens a clinic for people like him in your neighborhood would you hope the local council would object?	1.23	0.64	1.30	0.66	−0.94	0.11
Is the cause of this sort of illness something passing down in the family?	2.07	1.21	1.83	0.91	1.82	0.22
Is his illness something he might have brought on himself?	1.81	1.09	1.66	0.94	1.22	0.15
Should the doctors only let him leave hospital on condition he goes to see them regularly?	3.23	1.05	3.35	1.06	−0.93	0.11
Do you think a sympathetic family and friends can stop him becoming ill again?	3.16	0.96	3.70	0.70	−5.17*	0.64
Will a sympathetic family be more help to him than regularly taking medicine?	3.63	0.82	3.48	0.85	1.53	0.18
If he becomes ill again do you think it would be better to call the police first rather than the doctor?	1.29	0.53	1.16	0.53	2.00*	0.25
Might he have any special powers (to heal, to predict future events, to cause illness)?	1.94	1.12	1.50	0.82	3.56*	0.45
Could this illness be caused by some spirits or an enemy harming him?	1.70	0.94	1.82	1.10	−1.01	0.12

* *P* < 0.05; (-) item rescored so that higher scores represent more stigma; *d* = Cohen's *d* (measure of effect size in SD units)

### Rural-urban differences in additional stigma indicators

Rural-urban differences in additional stigma indicators are shown in the bottom panel of Table 2. These items were not included in the composite stigma score either because there was no clear a priori hypothesis predicting the direction of the item's relation to overall stigma or because the item was not consistently associated with overall stigma in the initial validation study.[14] Individuals in the urban sample were significantly more likely to believe that sympathetic others could help to prevent relapse, whereas individuals in the rural sample were more likely to believe that it is better to call the police rather than the doctor in the case of acute illness and, also, that the person might have special powers “to heal, to predict future events, to cause illness.”

Correlations between the additional stigma indicators and the composite stigma score are shown in Table 3; the third column identifies significant rural-urban differences in the magnitude of the correlation coefficients, calculated using Fisher's *Z*. Not wanting to work with a mentally ill person was highly correlated with overall stigma in the urban sample but showed no such relation in the rural sample. As well, the belief in special powers “to heal, to predict future events, to cause illness” was associated with more overall stigma in the urban sample but less overall stigma in the rural sample. In both samples, belief in the benefits of a sympathetic family, together with regular medication, is associated with fewer stigmas; whereas belief that the illness is caused by spirits is associated with more stigma.

**Table 3:** Comparison of rural and urban samples on relations between additional stigma indicators and composite stigma score

Stigma questions	Rural	Urban	Comparison
Would you be content if he was to work together with you in your workplace?	0.04	−0.54*	*
(If you do not have a job, answer as if you did.)			
If your local hospital opens a clinic for people like him in your neighborhood would you hope the local council would object?	−0.16	0.14	
Is the cause of this sort of illness something passing down in the family?	0.14	0.37*	
Is his illness something he might have brought on himself?	0.16	0.23*	
Should the doctors only let him leave hospital on condition he goes to see them regularly?	−0.06	0.24*	
Do you think a sympathetic family and friends can stop him becoming ill again?	−0.14	−0.24*	
Will a sympathetic family be more help to him than regularly taking medicine?	−0.30*	−0.23*	
If he becomes ill again do you think it would be better to call the police first rather than the doctor?	0.02	0.27*	
Might he have any special powers (to heal, to predict future events, to cause illness)?	−0.33*	0.27*	*
Could this illness be caused by some spirits or an enemy harming him?	0.20*	0.26*	

* A significant difference (*P* < 0.05) exists between rural and urban correlations with the composite stigma score

## DISCUSSION

Rural Indians showed a more stigmatizing attitude towards severe mental illness. Contrary to expectation, this study shows greater stigma and a punitive attitude amongst rural Indians as compared to urban Indians, especially amongst rural manual workers. This is at odds with literature that indicates a more benign attitude among rural populations towards persons with severe mental illness.[2,4,11,16,17] A few studies, however, argue the opposite[12,13,18] or report variable findings.[19,20] Strikingly, the highest levels of stigma were observed among rural manual laborers, an effect that remained even when education was controlled.

Although the composite stigma score was significantly higher amongst rural Indians compared to the urban sample, there are important variations and contradictions on response to individual items of the stigma questionnaire. These include rural subjects reporting a supportive tolerant attitude as indicated by a favorable response to living next door, not covering up the illness and expecting a full return to normality in the community. Yet the rural Indian sample seemed to lack faith in biomedical interventions and avoid specific inter-personal interactions relating to food, marriage and allow the vignette subject to be involved in an educational role. It might appear that rural Indian responses are more aligned with a bi-phasic model, which is characterized by an initial tolerant response to low levels of deviance, followed by sudden shift towards intolerance to high levels of deviance.[21] Although this study did not specifically collect data on availability of local biomedical help and treatment in each of the study sites, a rapid shift in the direction of “intolerance to severe mental illness” could well be due to availability of local services. These might include geographical or culturally inaccessible mental health services at the Kanpur and Kaziranga rural sites. This hypothesis can be tested by modifying the test vignette to frame questions that reflect differing stages of the illness, including perception of local mental health services.

Interestingly, unacceptability of severely mentally ill subjects at work showed a significant correlation with overall stigma in the urban sample but bore no relationship with stigma amongst the rural respondents. It is plausible that stigmatizing attitudes are common in rural agricultural societies but that these societies, at the same time, provide meaningful social and occupational roles for individuals with mental illness.[2] Several researchers have argued that illness recovery is contingent on local political economy.[2,16] This may well be a key determinant in shaping better outcome in low-income countries. For example, urban industrialized societies might encourage more benign attitudes towards the mentally ill while simultaneously denying them meaningful social roles. Indeed, such anthropological studies have argued that poor recovery from severe mental illness is more likely to be associated with demanding, specialized and rigid occupational roles; increased competition in urban industrialized capitalist economies and associated employment-labor dynamics.[16]

Findings from our Kolkata urban respondents, who generally opted for a more liberal attitude towards the subject in our vignette, require further contextualization. Their responses to the vignette might reflect social tolerance that could well be unique to a Bengali cultural notion of a romanticized, eccentric and meaningful label for madness that permits greater empathy.[22] In this context, ethnographic studies have argued for certain traditions that might place a positive value on empathy with severely mentally ill. Concepts such as *pagol* (“madness”) amongst Indian Bengalis are not analogous with western connotations of psychosis or schizophrenia.[23] However, such ethnographies[22] were carried out two decades ago, and it is unclear if these ideas have changed over time. Extending future study sites to include other Indian urban centers outside of West Bengal would permit testing hypotheses relating to both the impacts of cultural change as well as social geographical variation on stigmatizing attitudes.

In addition to the unexpectedly high stigma towards severe mental illness amongst rural Indian subjects, findings from the present study challenge the industrialization hypothesis at face value. The lack of a link between stigma and work attitudes in the rural sample is a significant finding. If industrialization is associated with increased stigma, which in turn has a negative impact on prognosis of severe mental illness, the study would have demonstrated more, not less, stigmatizing attitudes amongst the urban Indian sample in comparison to the rural. Our findings are in the reverse direction. However, there is a significant caveat to this logic. As discussed earlier, a meaningful occupation of affected subjects has been postulated to be an important determinant in shaping recovery. Our study findings offer indirect support for this premise as urban Indians, not rural, showed a strong link between stigma and not wishing to work with a mentally ill individual despite lower scores on stigma related to other aspects of social life. In other words, a meaningful occupation might override other social and interpersonal dimensions of stigma. Such complexities challenge a simple industrialization hypothesis and require further research.

The present study was based on an ethnographically derived, vignette-based questionnaire aimed primarily at capturing relatively static stigmatizing attitudes. Our experience cautions further research that relies on paper-pencil checklist or attitudinal questionnaires to examine a complex topic such as stigma. Additional ethnographic research based on participant observation would complement this approach by enhancing our understanding of how these attitudes are enacted in everyday social life. This would clarify what people do as opposed to what people say.

In conclusion, this study marks the first reported investigation deploying an ethnographically derived, psychometrically robust and cross-culturally validated instrument to test the industrialization hypothesis. Rural Indians showed more stigmatizing attitudes towards severe mental illness. Urban Indians reported a more liberal and tolerant attitude but were also more excluding of those with mental illness at work. Prospective longitudinal research is vital to examine the independent role of stigma and occupation in predicting outcome of severe mental illness. This study also opens the way for further combined quantitative epidemiological and qualitative ethnographic studies focusing on low-income countries during periods of economic and social change.

## APPENDIX: STIGMATIZATION QUESTIONNAIRE[5,14]

Here is a short account of a person who became ill. Please answer the questions about him:

This young man is twenty years old. He is not married and lives with his parents. He is friendly and hard working. He works in a local factory. One day he becomes ill and starts imagining things that are not true. He cannot do his job properly and eventually loses it. He spends a lot of time by himself. He hears people talking to him when there is no one there. His parents now become anxious but he does not get better. He starts shouting at the voices which he hears and he tells his family that they themselves are trying to hurt him. On one occasion he hits his father.

The family members are very distressed and frightened and do not know what is happening. They ask their neighbors: nobody thinks this is any sort of religious experience. The family members take him to the local doctor who tells them the young man is ill and gives him some tablets. The tablets do not help him. He does not eat properly. He seems puzzled by what is happening. He does not dress himself properly and is often dirty. He wanders about and says embarrassing things to people whom he meets in the streets.

His parents do not know what he is talking about. His doctor sends him to hospital, where he stays for two months. He gets better on some new tablets but still needs to take them when he leaves hospital. He does not hear the voices anymore, nor does he have the strange ideas, but he is very quiet and stays alone for much of the time. He occasionally talks to himself but is usually polite to his family. He goes often to see his doctor to get his tablets and wishes to go back to work.

Here are some questions about this person. Each one must be answered by whether you agree with the question:

Yes, very much Yes, a little No, not much No, not at all.

Put a tick in the appropriate box for each question. Remember this is not a test of knowledge but about how you really feel personally.

1. Would you be frightened if this man came to live next door to you?
2. Would you be content if he was to work together with you in your workplace? (If you do not have a job, answer as if you did.)
3. Do you think he will get ill again even if he takes the doctor's medicine?
4. Should he take part in meetings of his family which are to make important decisions?
5. Would you be happy if he married your sister?
6. Could he suddenly become physically violent?
7. If he was your brother would it be important not to let other people know that he had been ill, to avoid shame for your family?
8. If your local hospital opens a clinic for people like him in your neighborhood, would you hope the local council would object?
9. Is the cause of this sort of illness something passing down in the family?
10. Should the doctors tell him not to have any children in case he passes the illness on to them?
11. Should the doctors have let him out of hospital?
12. Is his illness something he might have brought on himself?
13. Should the doctors only let him leave hospital on condition he goes to see them regularly?
14. Do you think a sympathetic family and friends can stop him becoming ill again?
15. Will a sympathetic family be more help to him than regularly taking medicine?
16. Would it be wise for this man to inherit his parents” property?
17. If he becomes ill again, do you think it would be better to call the police first rather than the doctor?
18. Would you be happy if this person became the teacher of your children?
19. Will he be able to return to a completely normal life?
20. Should he stay in hospital for his whole life?
21. Would you eat food which he has cooked?
22. Would you avoid talking to him if possible?
23. Might he have any special powers (to heal, to predict future events, to cause illness)?
24. Could this illness be caused by some spirits or an enemy harming him?
25. Has any person you know personally ever had a similar illness? Yes/No
26. Could you give a name to this illness?
